# Lactic Acid Bacteria Isolated from Bovine Mammary Microbiota: Potential Allies against Bovine Mastitis

**DOI:** 10.1371/journal.pone.0144831

**Published:** 2015-12-29

**Authors:** Damien S. Bouchard, Bianca Seridan, Taous Saraoui, Lucie Rault, Pierre Germon, Candelaria Gonzalez-Moreno, Fatima M. E. Nader-Macias, Damien Baud, Patrice François, Victoria Chuat, Florian Chain, Philippe Langella, Jacques Nicoli, Yves Le Loir, Sergine Even

**Affiliations:** 1 UMR 1253 STLO, INRA, Rennes, France; 2 UMR1253 STLO, Agrocampus Ouest, Rennes, France; 3 Departamento de Microbiologia, ICB, Universidade Federal de Minas Gerais, Belo Horizonte, Brazil; 4 UMR 1282 Infectiologie et Santé Publique, INRA, Nouzilly, France; 5 CERELA-CONICET, Centro de Referencia por los Lactobacillus, San Miguel de Tucumán, Argentina; 6 Genomic Research Laboratory, Geneva University Hospital, Geneva, Switzerland; 7 INRA-MICALIS, Jouy-En-Josas, France; University Paris South, FRANCE

## Abstract

Bovine mastitis is a costly disease in dairy cattle worldwide. As of yet, the control of bovine mastitis is mostly based on prevention by thorough hygienic procedures during milking. Additional strategies include vaccination and utilization of antibiotics. Despite these measures, mastitis is not fully under control, thus prompting the need for alternative strategies. The goal of this study was to isolate autochthonous lactic acid bacteria (LAB) from bovine mammary microbiota that exhibit beneficial properties that could be used for mastitis prevention and/or treatment. Sampling of the teat canal led to the isolation of 165 isolates, among which a selection of ten non-redundant LAB strains belonging to the genera *Lactobacillus* and *Lactococcus* were further characterized with regard to several properties: surface properties (hydrophobicity, autoaggregation); inhibition potential of three main mastitis pathogens, *Staphylococcus aureus*, *Escherichia coli* and *Streptococcus uberis*; colonization capacities of bovine mammary epithelial cells (bMEC); and immunomodulation properties. Three strains, *Lactobacillus brevis* 1595 and 1597 and *Lactobacillus plantarum* 1610, showed high colonization capacities and a medium surface hydrophobicity. These strains are good candidates to compete with pathogens for mammary gland colonization. Moreover, nine strains exhibited anti-inflammatory properties, as illustrated by the lower IL-8 secretion by *E*. *coli*-stimulated bMEC in the presence of these LAB. Full genome sequencing of five candidate strains allowed to check for undesirable genetic elements such as antibiotic resistance genes and to identify potential bacterial determinants involved in the beneficial properties. This large screening of beneficial properties while checking for undesirable genetic markers allowed the selection of promising candidate LAB strains from bovine mammary microbiota for the prevention and/or treatment of bovine mastitis.

## Introduction

Bovine mastitis is an inflammation of the mammary gland and most often results from bacterial infection. These intramammary infections cause huge economic losses in the dairy farming sector and dairy industry [1,2]. To date, the treatment of bovine mastitis is predominantly based on antibiotics. However, they are not totally effective and contribute to the emergence and transmission of antibiotic resistance within the host microbiota, which include both commensals and opportunistic pathogens. There is thus a need for alternative strategies that can be used as prophylactic or alternative or complementary curative treatments.

One alternative is the emerging concept of mammary probiotics. For this purpose, lactic acid bacteria (LAB) are good candidates due to their Generally Recognized As Safe (GRAS) status and their recognized technological and inhibitory properties. LAB have been investigated for many years for their beneficial effects on human health [3,4]. They contribute to maintaining the balance of natural microbiota (i.e., vaginal and gut), by competing with pathogens for tissue colonization, modulating virulence expression or stimulating the innate immune system [5,6]. Probiotics have also been shown to be an effective alternative to antibiotherapy for the treatment of human mastitis [7].

Likewise, the use of probiotics has gained interest in the veterinary community. The autochthonous bovine mammary microbiota was investigated, either by isolation on selective culture media or by 16S rRNA sequencing to identify microorganisms with inhibitory properties against mastitis pathogens [8]. A bacteriocin-producing *Lactococcus lactis* was shown to be as effective *in vivo* as a conventional antibiotic treatment to treat cow mastitis [9]. Encouraging results were also obtained *in vitro* with a strain of *Lactobacillus perolens*, which was shown to inhibit several mastitis-causing pathogens as a result of its coaggregation with mastitis pathogens and its capacity to colonize bovine mammary epithelial cells (bMEC) [10]. When used in *in vivo* intramammary injections, this strain did not show adverse effects on mammary tissue [11]. Similarly, we recently demonstrated *in vitro* that different *Lactobacillus casei* strains, including one strain isolated from the bovine teat canal, inhibit adhesion and internalization of *S*. *aureus* within bMEC without affecting the bMEC physiology [12].

Based on these observations, the objective of this study was to isolate LAB from bovine mammary gland microbiota and to characterize their beneficial properties in order to select good candidates to be included in a mammary probiotic cocktail against infectious mastitis. As beneficial properties, we first evaluated LAB capacities to inhibit growth of the three main pathogens associated with mastitis, i.e., *Staphylococcus aureus*, *Escherichia coli* and *Streptococcus uberis*, through acidification and production of hydrogen peroxide and bacteriocin-like compounds [13]. Secondly, we characterized their surface properties including their autoaggregation capacities and their degree of hydrophobicity, which have been associated with the ability to colonize host tissues [14]. Thirdly, we investigated LAB capacities to adhere to bMEC (MAC-T cell line) in order to estimate their potential to colonize the mammary gland epithelium *in vivo* and, as a consequence, to compete with pathogens for tissue colonization. Finally, their ability to stimulate the innate immune system was estimated by measuring their capacity to modulate production of a pro-inflammatory cytokine (IL-8) by the bMEC line PS. IL-8 is involved in the first steps of the inflammatory response of the mammary gland, leading to neutrophil recruitment [15]. The full sequencing of five out of ten strains was included so as to identify potential genomic determinants of the colonization and immunomodulation capacities and to check for undesirable or unfavorable genetic elements, e.g., antibiotic resistance determinants. This characterization allowed us to identify promising LAB strains that exhibited a good potential to colonize the mammary gland ecosystem, as well as immunomodulation properties.

## Materials and Methods

### Sampling

The samples were collected from 20 Holstein dairy cows in two herds belonging to the InterBioBretagne network (organic farming organization), in the Brittany region of France. One quarter per cow was sampled, corresponding to the left or right rear quarter. Only quarters without any clinical symptoms of mastitis were selected. Teats were thoroughly washed with water and cleaned with 70% ethanol and individual paper towels. Teat canals were then sampled in two different ways. A 5-mm sterile Histobrush® swab (D. Dutscher, Brumath, France) was inserted 5 mm inside the teat apex and turned three times before removal. The swabs were immediately placed in tubes containing 2 mL of sterile peptone solution (20 g/L peptone; 5 g/L sodium chloride). Foremilk samples were then collected in sterile plastic tubes. All samples were stored on ice until processing in the laboratory.

The protocol was reviewed and approved by the Regional Ethics Committee for Animal Use and Care (Bretagne, France). Sampling is part of a classical veterinary practice. According to the European directive 2010 / 63 / EU, this type of experiment does not require an authorization request. All persons involved in sampling the cows used in this study were licensed veterinarians. All procedures were part of routine care or surveillance. Permission was received from the owners of the cattle to conduct and publish this research. No animals were sacrificed for the purpose of this study.

### Isolation of LAB strains

Foremilk samples were homogenized with nine volumes of a trisodium citrate solution (2% w/v) and centrifuged (6650 g/5 min/4°C). The pellet was then suspended in 2 mL of sterile peptone solution. Bacterial suspensions corresponding to swab and foremilk samples were grown on M17, MRS or MRS 5.4 by diluting 100 μL of bacterial suspension in 12 mL of M17, MRS at pH 6.8 and MRS broth acidified at pH 5.4 (hereafter referred to as MRS 5.4; more selective for lactobacilli), followed by a 48-h incubation at 37°C in an anaerobic jar, for selective cultivation. Serial dilutions of the enriched bacterial suspensions were then performed, plated on M17, MRS and MRS 5.4, and incubated for 48 h at 37°C in an anaerobic jar. Colonies with different morphotypes were isolated and set in collection in the enrichment medium supplemented with 15% glycerol and stored at -80°C.

### Genetic identification of isolates

Each isolate was identified by sequencing the 16S rDNA gene. Genomic DNA was isolated from a 2-mL overnight culture on M17, MRS or MRS 5.4 after centrifugation (6000 g/5 min/4°C), washing with 1 mL of peptone solution, and an additional centrifugation (6000 g/5 min/4°C). The pellet was lysed for 45 min at 37°C in 180-μL lysis buffer containing 20 mM Tris-HCl (pH 8), 2 mM EDTA, 1% triton X100 and 20 g/L lysozyme (MP Biomedicals, Illkirch, France). Genomic DNA was purified using the DNeasy® Blood & Tissue Mini Kit (Qiagen, Courtaboeuf, France), according to the manufacturer’s recommendations.

PCR amplification of 16S rDNA was performed using a Veriti™ 96-well thermal cycler (Applied Biosystems, Foster City, CA, USA) in a 50-μL final volume containing 20 ng genomic DNA, 1x HF Phusion buffer, 0.5 μM of primers W001 (5’-AGAGTTTGATCMTGGCTC) and W002 (5’-GNTACCTTGTTACGACTT)[16], 200 μM dNTP and 1U Phusion polymerase (New England Biolabs). The PCR conditions were as follows: denaturation step at 95°C for 5 min, followed by 30 cycles of denaturation at 95°C for 30 s, annealing at 50°C for 30 s, and extension at 72°C for 1 min 30 s. A final extension step was performed for 10 min at 72°C. Sequencing of the PCR product was performed by LGC Genomics (Berlin, Germany) using the same primers. Sequences were further compared to Genbank Database using BLAST. Identification was achieved at specie level for similarity higher that 98%. When similarity search gave the same score with several species, isolates were identified at the genus level.

The 22 LAB strains retained for PFGE analysis (see results) have been registered in the collection of the CIRM-BIA Biological Resource Center (Rennes, France).

### Characterization of LAB isolates by pulse-field gel electrophoresis

The PFGE molecular fingerprints of LAB isolates were obtained using the method adapted from Smith and Cantor [17]. The culture and the agarose blocks were prepared as previously described [18]. The blocks were equilibrated for one hour in a restriction buffer at 4°C and transferred to 300 μL of fresh digestion buffer containing 15 U of *Sma*I or 25 U of *AscI* endonucleases (New England Biolabs, Hitchin, UK). The blocks were incubated for 4 h at 25°C for *Sma*I and at 37°C for *AcsI*. PFGE was carried out with a CHEF-DR II apparatus (Bio-Rad, Australia) in a 1% agarose gel (w/v) (Ultrapur, Gibco-BRL, Scotland) in 0.5 × TBE at 200 V and at 14°C with the following pulse times and total running time: *Sma*I (initial time—2 s; final time—20 s; total running time—20 h), *AscI* (2 s; 20 s; 21 h). After electrophoresis, gels were stained with GelRed and visualized under UV light. Photographs of PFGE gels were scanned and the band profiles were analyzed using BioNumerics, version 4.1 (Applied Maths, Kortrijk, Belgium). Comparisons between the normalized band profiles were made using the Dice similarity coefficient with an optimization of 1%. Clustering of strain profiles was accomplished using the unweighted pair group method with arithmetic averages (UPGMA).

### Production of hydrogen peroxide

Screening of hydrogen peroxide production by the isolates was performed using a TMB (3, 3’, 5, 5’-tetramethylbenzidine) assay, as previously described [8]. Strains were classified according to the color intensity as non-producers, low producers or high producers of H_2_O_2_.

### Production of antagonistic substances

The screening of the antimicrobial potential of the LAB supernatants was carried out using the agar plate diffusion method as previously described [19]. Supernatants were either native, neutralized with NaOH or neutralized and treated with 1000 U/mL of catalase (Sigma Aldrich, USA) for 1 h at 25°C. Six pathogenic strains corresponding to the main species responsible for bovine mastitis were used as indicators: *Staphylococcus aureus* RF122 and Newbould 305 (N305), which were isolated from bovine mastitis [20,21], *Streptococcus uberis* LMA1675 and LMA1672, and *Escherichia coli* LMA1678 and LMA1674. *S*. *uberis* and *E*. *coli* strains were isolated from the mammary ecosystem during this study.

### Bacterial surface properties

Autoaggregation capacities (i.e., the capacity of a strain to form aggregates in a bacterial suspension) were determined as previously described [8]. The degree of hydrophobicity was evaluated using the Microbial Adhesion To Hydrocarbons (MATH) method with hexadecane (Sigma Aldrich, US) [8]. Strains were classified as low, medium and high, according to their hydrophobicity or autoaggregative capacities.

### Adhesion and internalization assays

The established bMEC MAC-T line (Nexia Biotechnologies, Quebec, Canada) was cultured in T75 cell culture flasks in DMEM containing 10% heat-inactivated fetal calf serum (FCS), 100 U/mL penicillin, 10 mg/mL streptomycin, and 5 μg/mL insulin (D. Dutscher). Cells were incubated at 37°C in a humidified incubator with 5% CO_2_. They were cultured to a confluent monolayer, treated with 0.05% trypsin (Gibco-BRL, Grand Island, NY, USA), and suspended in fresh MAC-T medium at a concentration of 2x10^5^ cells/mL. For adhesion and internalization assays, cells were then seeded in 12-well plates (2x10^5^ cells/well) and incubated overnight at 37°C in 5% CO_2_ to obtain a confluent monolayer.

#### Adhesion assay

Adhesion assays were performed at least three times, as previously described [12]. Briefly, confluent monolayers of MAC-T cells (2.5x10^5^ cells/well) were washed twice with PBS and incubated at 37°C in 5% CO2 with 1 mL of LAB suspensions in DMEM at 1x10^8^ cfu/mL or 5x10^8^ cfu/mL to achieve a multiplicity of infection (MOI) of LAB organisms to cells of 400:1 and 2000:1, respectively. LAB adhesion was measured 1 h post-interaction. After washing four times with PBS, the monolayer was treated with 0.05% trypsin, centrifuged for 5 min at 800 g and lysed with 0.01% triton. The population of LAB that adhered to the cells was determined by colony counting, on M17 agar for lactococci and MRS agar for lactobacilli, from serial dilutions of the cell lysates.

#### Internalization assays

Internalization assays were performed at least three times in the same conditions as adhesion assays (same MOI) except that LAB internalization was measured 2 h post-interaction. Following incubation with LAB, cells were washed four times with PBS and incubated for 2 h with 1 mL of DMEM containing 100 μg/mL gentamicin in order to kill extracellular bacteria and allow the numeration of the internalized bacterial population only. Subsequently, cells were lysed and the population of internalized LAB was determined as described above.

### Immunomodulation properties of LAB strains on the PS cell line

The immunomodulation properties of LAB strains were evaluated by measuring the production of the pro-inflammatory cytokine IL-8, a cytokine involved in the first step of the inflammatory response, in the newly described PS bMEC line [22]. The MAC-T cell line barely modifies its interleukin gene expression pattern when stimulated with pathogens, whereas the PS cell line was shown to significantly react to stimulation, as illustrated by the secretion of IL-8 in the presence of *E*. *coli* [22]. The PS cell line (INRA, Tours, France) was cultured at 37°C in a humidified chamber with 5% CO_2_, in DMEM/F12 advanced medium (D. Dutcher) containing 10 mg/mL of IGF-1 (Peprotech), 5 ng/mL of FGF (Peprotech), 5 ng/mL of EGF (Sigma-Aldrich), 1 μg/mL of hydrocortisone (Sigma-Aldrich), 20 mM of Hepes buffer (D. Dutcher) and 2 mM of glutamine (Gibco).

Stimulation of PS cells was performed with LAB alone or with LAB in the presence of *E*. *coli*, which is known to stimulate IL-8 production. Subsequent measurements of IL-8 production were essentially performed as previously described [22]. PS cells were seeded at 2x10^4^ cells/well in a 96-well plate and incubated for 72 h at 37°C (until cells formed a confluent layer). Cells were then cultured overnight (16 h) in fresh stimulating medium without growth factors (PS stimulation medium). Cells were then washed twice with HBSS (Hank’s Balanced Salt Solution; Sigma-Aldrich, Saint-Quentin Fallavier, France) and stimulated with either LAB at a MOI of 100:1 or *E*. *coli* P4 at a MOI of 1:1, or both species. Cells were incubated for 3 h, washed with HBSS and incubated in PS stimulation medium supplemented with 10 μg/mL of gentamicin for 21 h, completing 24 h of infection. After this incubation, the supernatant was collected and stored at -20°C until use. Concentration of IL-8 in the supernatant was measured by ELISA as previously described [22]. All samples were analyzed in duplicate in two independent assays (a total of four data points). IL-8 production by PS cells alone was used as a reference to assess the effect of LAB alone, whereas IL-8 production by *E*. *coli*-stimulated PS cells was used as a reference to assess the effect of LAB in the presence of *E*. *coli*. Data were normalized relative to these references.

### Immunomodulation effects of LAB strains on HT-29 cell line

Experiments were performed as previously described [23]. Briefly, experiments were initiated when HT-29 cells were at confluence (∼1.83×10^6^ cells/well). LAB were added at MOI 40:1 in 50 μL DMEM in a total volume of 500 μL. Cells were stimulated simultaneously with recombinant human TNF-α (5 ng/mL; Peprotech, NJ, USA) for 6 h at 37°C in 10% CO_2_. Stimulation of the HT-29 cell line with TNF-α alone was used as the reference condition. All samples were analyzed in triplicate in three independent assays (a total of nine data points). After coincubation, cell supernatants were collected and mixed with anti-protease cocktail, as indicated by the manufacturer (Complete, EDTA-free tablets, Roche) and frozen at −80°C until further analysis of IL-8 concentrations by ELISA (Biolegend, San Diego, CA, USA).

### Genome sequencing

Genomic DNA of *L*. *brevis* 1595, *L*. *casei* 1542, *L*. *lactis* 1596, and *L*. *plantarum* 1610 and 1612 was extracted and purified as described above. Genome sequencing, assembly and annotation were performed as previously described [20]. Briefly, paired-end sequencing was performed using HiSeq sequencing system (Illumina, San Diego, USA). Sequencing statistics are included in S1 Table. Coding sequence (CDS) detection was performed with the Glimmer3 software application [24]. Gene products were subjected to protein location prediction using the SurfG software package [25]. Genomes were further screened using the CD-search tool of the Conserved Domain Database for the presence of specific domains [26]. In particular, all genomes were screened for the presence of domains involved in binding to mucin, collagen and fibronectin: pfam06458 (MucBP, mucin-binding protein domain), cl05785 (MucBP superfamily), pfam5737 (Collagen_bind, collagen-binding domain), pfam05738 (Cna-B, Cna protein B-type domain;this domain is found in the *Staphylococcus aureus* collagen-binding surface protein, but does not mediate collagen binding), cl15753 (collagenBindB superfamily), cl05349 (collagen_bind superfamily), pfam07299 (FBP, fibronectin-binding domain), pfam05833 (FbpA, fibronectin-binding protein A N-terminus), cl06363 (FBP superfamily), pfam00497 (SBP-bac-3; bacterial extracellular solute binding proteins, family 3). Systematic analysis of the conserved domain content of proteins annotated as Internalin was also included.

In addition, all genomes were screened for the presence of genes potentially encoding antibiotic resistance using the annotation tool of the Antibiotic Resistance Genes Database (ARDB; http://ardb.cbcb.umd.edu/blast/genome.shtml). Genome sequences have been deposited at DDBJ/EMBL/GenBank under the accession numbers LDEI00000000 (*Lactobacillus brevis* CIRM-BIA 1595), LDEJ00000000 (*Lactobacillus casei* CIRM-BIA 1542), LDEK00000000 (*Lactococcus lactis* CIRM-BIA 1596), LDEL00000000 (*Lactobacillus plantarum* CIRM-BIA 1610), LDEM00000000 (*Lactobacillus plantarum* CIRM-BIA 1612).

### Statistical analysis

Statistical analysis was performed using R software [27]. The differences in adhesion and internalization capacities among strains were assessed using one-way analysis of variance. Strains were then grouped using Tukey’s range test. The capacity of LAB to modulate IL-8 production by PS and HT-29 cells was assessed using the Mann-Whitney test.

## Results

### Isolation and identification of lactic acid bacteria within the bovine mammary ecosystem

Sampling the teat canals of 20 cows on two farms led to the isolation and identification of 165 isolates. To avoid redundancy, a selection of these isolates was carried out considering only one clone per species and per cow, ending up with a panel of 76 isolates. These isolates mainly corresponded to LAB, and included enterococci (28.9%), streptococci (28.9%), lactobacilli (22.4%), and lactococci (6.6%). The remaining isolates corresponded to enterobacteria (9.2%) and staphylococci (3.9%), which are not inhibited on the elective media used (S2 Table). Identification of these isolates based on 16S RNA analysis revealed that streptococcal isolates mainly corresponded to species commonly associated with bovine mastitis (*S*. *infantarius* and *S*. *uberis*) [1] and that enterococcal isolates are common fecal flora. We thus focused on the 22 isolates belonging to *Lactococcus* and *Lactobacillus* genera, which include one *Lactococcus lactis*, four *Lactococcus garvieae*, four *Lactobacillus brevis*, 11 *Lactobacillus plantarum* and two *Lactobacillus casei* isolates. At this step, the probiotic strain *L*. *casei* BL23 was also added to the panel as a reference strain since this strain was previously shown to exhibit probiotic properties [28] and we recently demonstrated that this strain was able to inhibit *S*. *aureus* internalization into bovine MEC [12].

The 22 LAB isolates and *L*. *casei* BL23 were then characterized by PFGE (S1 Fig). All four *L*. *garvieae* isolates belonged to the same cluster and, similarly, the two *L*. *casei* isolates had the same PFGE profile. The 11 *L*. *plantarum* isolates fell into seven groups with unique PFGE profiles and the four *L*. *brevis* isolates corresponded to three unique PFGE profiles. Characterization of the beneficial properties was then done on ten arbitrarily selected isolates corresponding to unique PFGE profiles (i.e., corresponding to unique strains), to avoid any risk of redundancy. The actual panel of strains thus includes one *L*. *lactis*, one *L*. *garvieae*, three *L*. *brevis*, four *L*. *plantarum* and one *L*. *casei* isolates (in addition to *L*. *casei* BL23, used as the control) (Table 1).

**Table 1:** Characterization of surface and antagonistic properties of LAB strains isolated from bovine teat canal.

Name		Sample	Surface properties				Antimicrobial properties			
		type	Hydrophobicity		Autoaggregation		H2O2 production	Acidification	Diffusion test	
Species	CIRM-BIA		% ^a^	Gr ^b^	% ^c^	Gr ^d^	TMB test	pH SN ^f^	native SN ^g^	neutralized SN ^g^
*Lactococcus lactis*	1596	Foremilk	21	L	10	L	NP ^**e**^	4.28	*S*. *aureus* RF122 / NB305, *E*. *coli* LMA1678 /LMA1674	-
*Lactococcus garvieae*	1605	Swab	7	L	7	L	NP	4.45	-	-
*Lactobacillus brevis*	1613	Foremilk	25	L	8	L	NP	5.25	-	-
*Lactobacillus brevis*	1595	Foremilk	46	M	15	L	NP	5.23	-	-
*Lactobacillus brevis*	1597	Swab	35	M	66	M	NP	5.09	-	-
*Lactobacillus plantarum*	1610	Foremilk	60	M	8	L	NP	3.89	*S*. *aureus* RF122, *E*. *coli* LMA1678/LMA1674, *S*. *uberis* LMA1675/LMA1672	-
*Lactobacillus plantarum*	1612	Foremilk	0	L	13	L	NP	3.84	*S*. *aureus* RF122, *E*. *coli* LMA1678/LMA1674, *S*. *uberis* LMA1675/LMA1672	-
*Lactobacillus plantarum*	1602	Foremilk	7	L	11	L	NP	3.93	*S*. *aureus* RF122, *E*. *coli* LMA1678/LMA1674, *S*. *uberis* LMA1675/LMA1672	-
*Lactobacillus plantarum*	1601	Swab	5	L	14	L	NP	3.92	*S*. *aureus* RF122, *E*. *coli* LMA1678/LMA1674, *S*. *uberis* LMA1675/LMA1672	-
*Lactobacillus casei* ^*n*^	1542	Swab	6	L	14	L	NP	4.10	*S*. *aureus* RF122, *E*. *coli* LMA1678/LMA1674, *S*. *uberis* LMA1675/LMA1672	-
*Lactobacillus casei BL23*			10	L	13	L	NP	4.17	*S*. *aureus* RF122, *E*. *coli* LMA1678/LMA1674, *S*. *uberis* LMA1675/LMA1672	-

^a^ Percentage of hydrophobicity

^b^ Strains were classified as low (L) or medium (M) according to their hydrophobicity capacities

^c^ Percentage of autoaggregation

^d^ Strains were classified as low (L) or medium (M) according to their autoaggregative capacities

^e^ NP: non producer

^f^ SN: supernatant

^g^ Indicator strains inhibited by LAB supernatants in an agar plate diffusion test; “-”indicates that none of the indicator strains were inhibited.

### Characterization of inhibitory potential against pathogenic bacteria

None of the tested strains was found to produce hydrogen peroxide, as measured by the colorimetric method on TMB agar plates (Table 1). Production of inhibitory compounds in the supernatant was tested using the agar plate diffusion method (see Materials and methods for details). Untreated supernatants of seven strains were able to inhibit, at least partially, the indicator strains. All *L*. *casei* and *L*. *plantarum* strains inhibited growth of all the indicator strains except that of *S*. *aureus* N305. On the contrary, no growth inhibition was observed for the *L*. *garvieae* and *L*. *brevis* strains tested. An intermediate inhibitory capacity was observed for *L*. *lactis* 1596. Inhibition was relieved in all cases when supernatants were neutralized with NaOH.

### Surface properties of LAB isolated from the bovine mammary ecosystem

A great majority of strains (eight out of eleven) exhibited a low hydrophobicity (MATH < 33%), whereas no strain was classified as highly hydrophobic (MATH > 66%). Three strains had medium hydrophobicity (33% < MATH < 66%), namely *L*. *plantarum* 1610 and *L*. *brevis* 1595 and 1597. Autoaggregation capacities were low for all strains but one, *L*. *brevis* 1597, which had a medium autoaggregation capacity.

### Colonization potential of LAB

**Fig 1:**
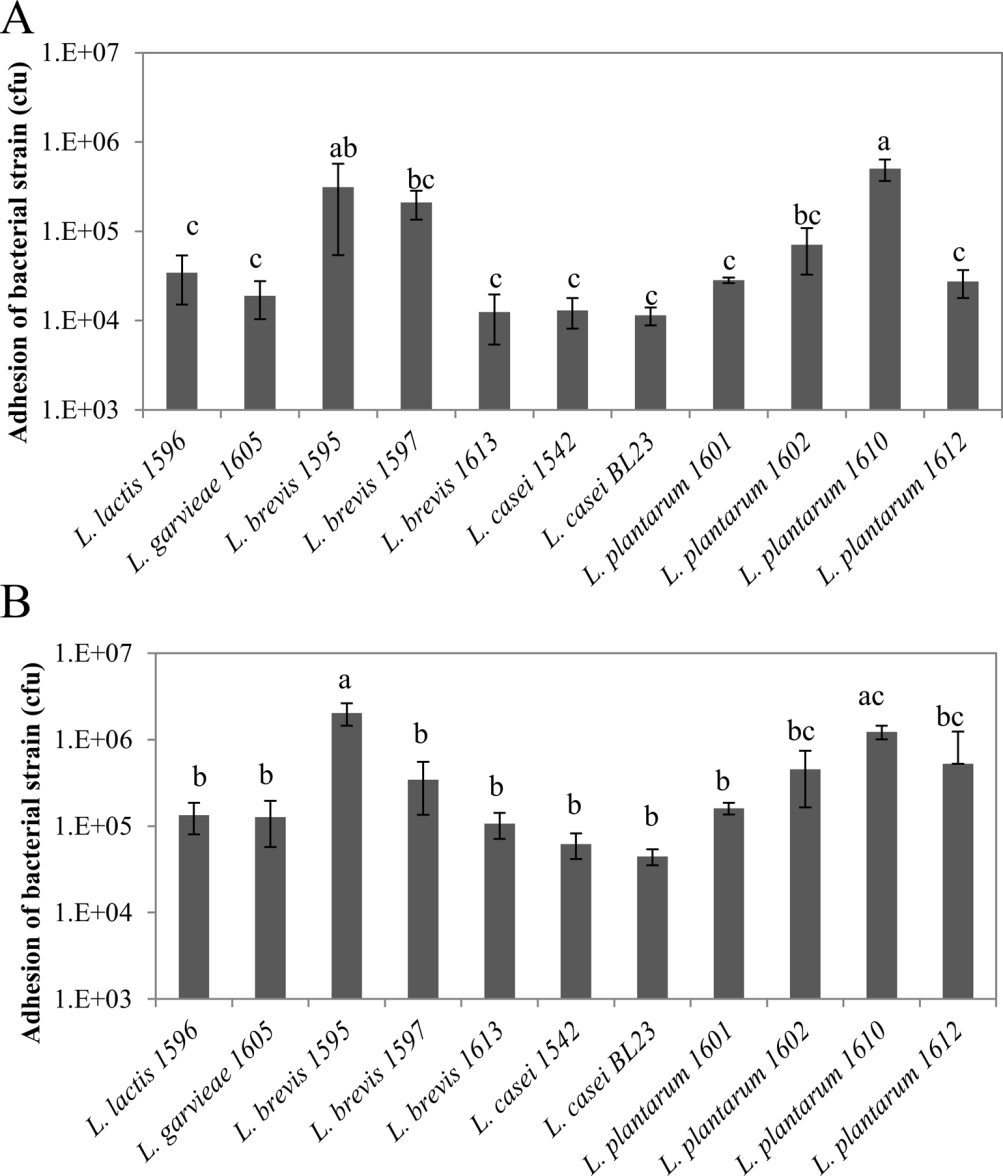
Adhesion of lactic acid bacteria to bovine mammary epithelial cells. Lactic acid bacteria populations that adhered to bMEC were determined after 1 h of interaction at a MOI of 400:1 (A) and 2000:1 (B), respectively. Data are presented as mean population per well (i.e., corresponding to 2.5x10^5^ bMEC) ± standard deviation. Each experiment was done in triplicate and differences between strains were assessed using a one-way analysis of variance, followed by Tukey’s range test. Letters a, b, c and d indicate homogeneous statistical processing groups that were significantly different according to Tukey’s range test.

Adhesion capacities of LAB were highly strain-dependent, with differences between strains of up to ~1.6 LOG_10_, independently of the MOI (Fig 1). Inter- and intra-species variability was observed as illustrated for *L*. *brevis* and *L*. *plantarum*. Hence, at a MOI of 400:1, the adhesion rate of *L*. *brevis* 1613 was 1.2x10^4^ cfu per well (corresponding to 2.5x10^5^ MAC-T cells), whereas it reached 3.1x10^5^ and 2.1x10^5^ cfu per well for *L*. *brevis* 1595 and 1597, respectively. Likewise, the adhesion rate of *L*. *plantarum* 1610 was 5x10^5^ cfu per well, whereas it was ~2.7x10^4^ cfu per well for *L*. *plantarum* 1601 and 1612. Two strains, *L*. *brevis* 1595 and *L*. *plantarum* 1610, exhibited adhesion capacities significantly higher than that of the others for a MOI of 400:1. These trends were confirmed at a MOI of 2000:1 but differences between strains were attenuated.

**Fig 2:**
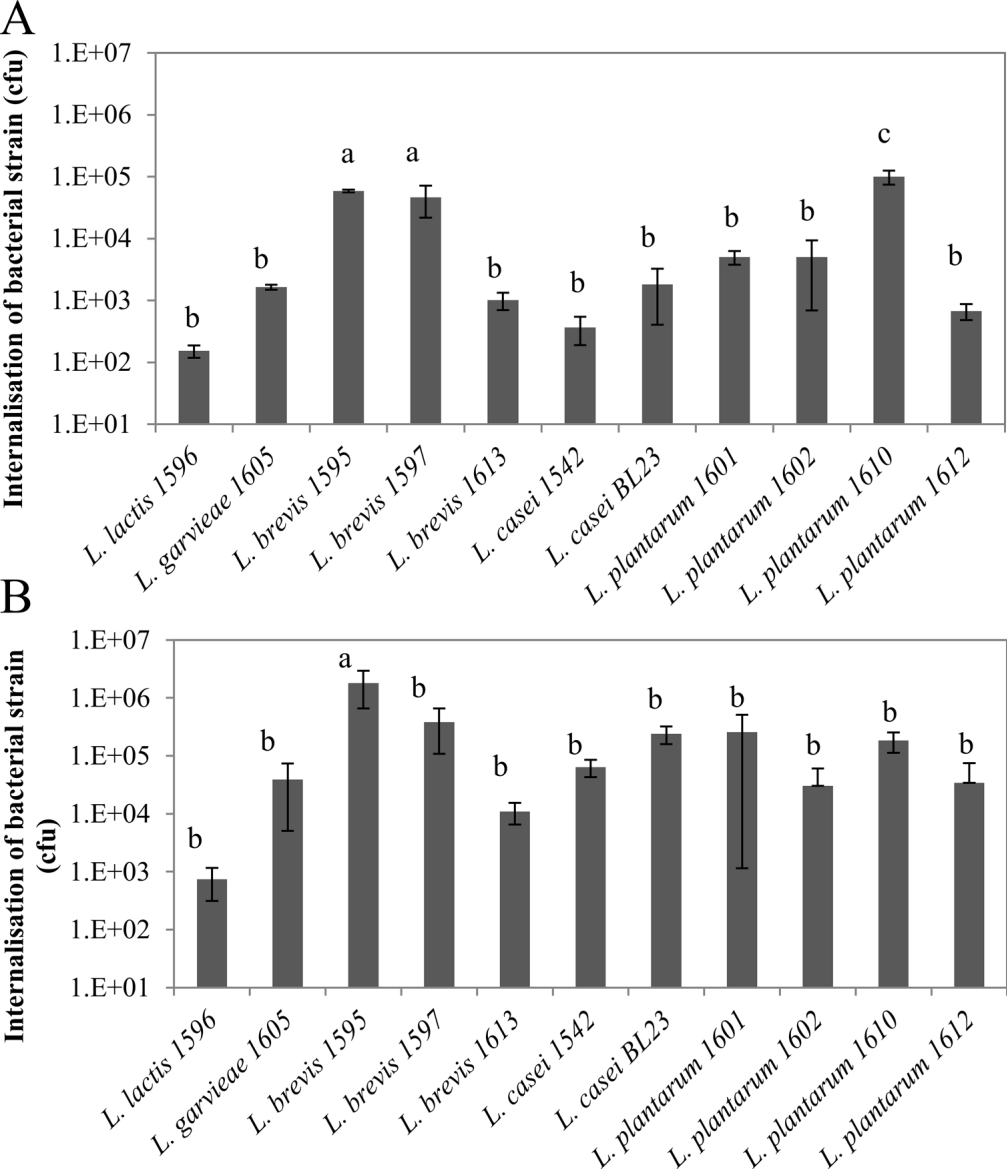
Internalization of lactic acid bacteria into bovine mammary epithelial cells. Lactic acid bacteria populations internalized into bMEC were determined after 2 h of interaction at a MOI of 400:1 (A) and 2000:1 (B), respectively. Data are presented as mean population per well (i.e., corresponding to 2.5x10^5^ bMEC) ± standard deviation. Each experiment was done in triplicate and differences between strains were assessed using one-way analysis of variance, followed by Tukey’s range test. Letters a, b, c and d indicate homogeneous statistical processing groups that were significantly different according to Tukey’s range test.

Internalization capacities of LAB were also highly strain-dependent, with differences of up to 3 LOG_10_, independently of the MOI (Fig 2). Three strains, *L*. *brevis* 1595 and 1597 and *L*. *plantarum* 1610, exhibited internalization capacities significantly higher than that of the others at a MOI of 400:1. Hence, internalization rates of *L*. *brevis* 1595, 1597 and *L*. *plantarum* 1610 at a MOI of 400:1 were 5.9x10^4^, 4.7x10^4^ and 1.0x10^5^ cfu per well, respectively, whereas it was between 1.5x10^2^ and 5.0x10^3^ cfu per well for the other strains. Differences were strongly attenuated at a MOI of 2000:1 since only *L*. *brevis* 1595 internalized at a significantly higher rate compared to the others.

Of note, the cellular layer integrity was not affected by incubation with any of the LAB in the conditions used: the MAC-T cells population remained constant at 2.5x10^5^ cells per well, and the cellular layer did not exhibit any changes in cell morphology during the experiment (not shown).

### Characterization of the immunomodulation potential of selected LAB

**Fig 3:**
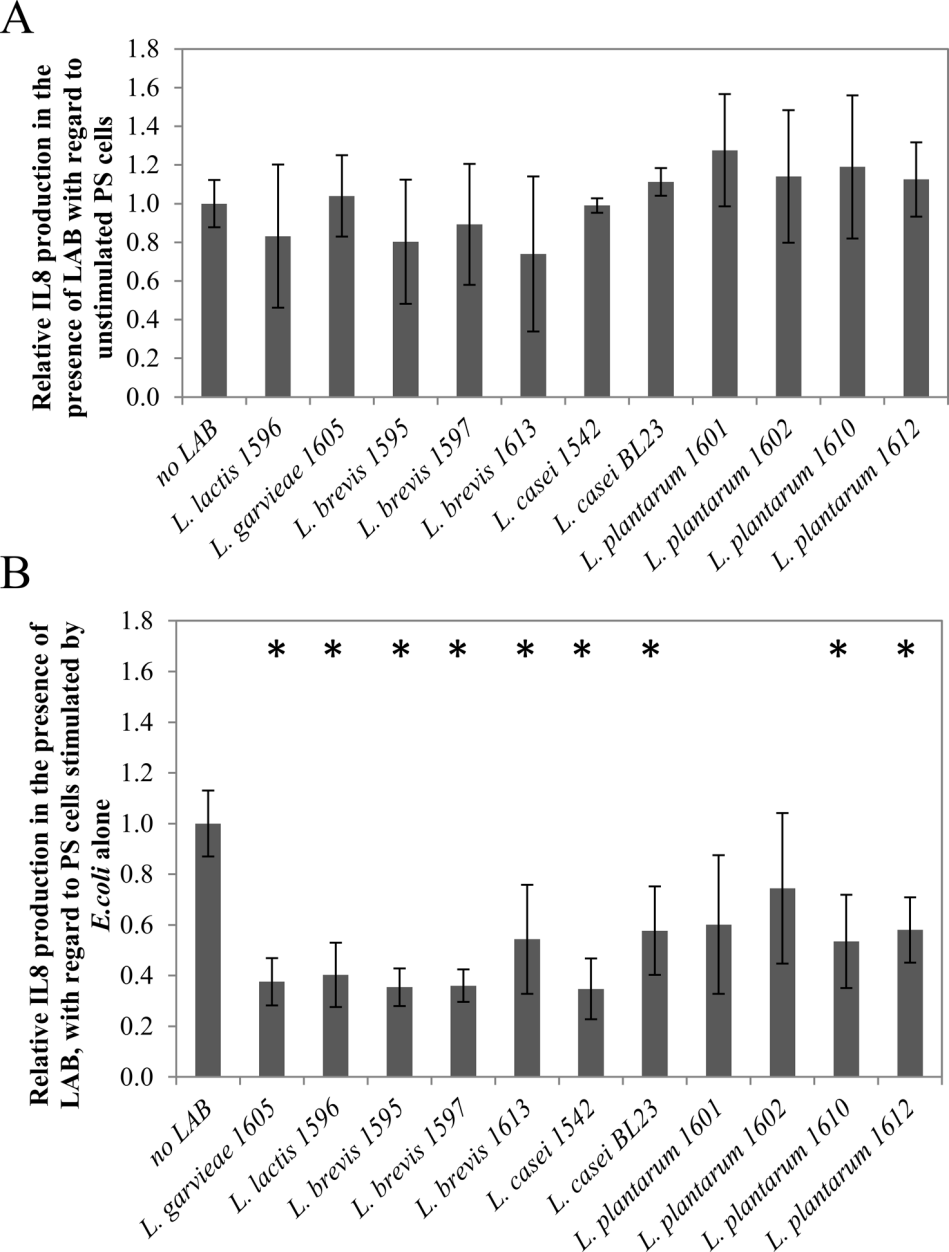
Modulation of cytokine IL-8 production by LAB isolates. A: modulation of IL-8 production by the PS cell line in the presence of LAB isolates (MOI 100:1). Bars represent the mean IL-8 production ± standard deviation for four assays (two biological and two technical replicates), normalized with regard to IL-8 production by unstimulated PS cells (76 +/-16 pg/mL as a mean); B: modulation of IL-8 production by the *E*. *coli*-stimulated PS cell line in the presence of LAB isolates. *E*. *coli* was used at MOI 1:1 and LAB at MOI 100:1. Bars represent the mean IL-8 production ± standard deviation for four assays, normalized with regard to IL-8 production by *E*. *coli*-stimulated PS cells (368 ± 88 pg/mL as a mean). Differences in IL-8 production with regard to the reference condition were assessed using the Mann-Whitney test (* p < 0.05).

The capacity of LAB to modulate the production of the pro-inflammatory cytokine IL-8 was assessed on the PS bMEC line by incubating either LAB alone with PS cells or LAB with *E*. *coli*-stimulated PS cells. LAB alone at a MOI of 100:1 did not significantly affect IL-8 secretion by PS cells (Fig 3). Immunomodulation properties of LAB occurred with *E*. *coli*-stimulated PS cells. Hence, all strains but two led to a significant decrease of IL-8 secretion by PS cells, with *L*. *brevis* 1595, 1597 and *L*. *casei* 1542 being the most efficient (2.8–2.9 fold decrease of IL-8 secretion, p_val_ < 0.05). Only *L*. *plantarum* 1601 and 1602 did not significantly affect IL-8 secretion by *E*. *coli*-stimulated PS cells.

In a first attempt to evaluate immunomodulation properties of LAB, screening of LAB was also done on the HT-29 model, which has been widely used to assess immunomodulation properties of LAB in the human gut context [23] (S2 Fig). Using this model, *L*. *garvieae* 1605, *L*. *casei* 1542 and, to a lesser extent, *L*. *brevis* 1595, were shown to slightly (1.1 to 1.4-fold), yet significantly, stimulate the production of IL-8 in HT-29 cells, compared to IL-8 production in the reference condition (HT-29 cells stimulated with TNF-α alone).

### Genome sequencing, identification of proteins potentially exposed at the cell surface and potential antibiotic resistance genes

The total genomes of five out of the ten LAB that were characterized, namely *L*. *brevis* 1595, *L*. *casei* 1542, *L*. *lactis* 1596 and *L*. *plantarum* 1610 and 1612, were determined, leading to the identification of 2429, 2760, 2339, 3091 and 3038 CDS, respectively. These strains were selected for diversity purposes since they represent almost all species (except *L*. *garvieae*) and exhibit different colonization and immunomodulation properties. *L*. *garvieae* was excluded at this step as its safety status may be questionable [29]. Combining annotations of proteins with their localization (PSE and secreted proteins) as well as the presence of specific conserved domains allowed us to establish a list of proteins potentially involved in tissue colonization through their binding to cells or an extracellular matrix (collagen, mucin, fibronectin) (S3 Table). All LAB strains, including BL23, encode three to six proteins containing a collagen-binding domain and one or two proteins containing a fibronectin-binding domain. All strains contain sortases, with up to four sortases for *L*. *casei* strains. On the contrary, strain-to-strain variations occurred in the genomic content for MucBP domains, with three to four proteins with a MucBP domain for *L*. *brevis* 1595 and *L*. *plantarum* strains, one for *L*. *lactis* 1596 and no protein with a MucBP domain in *L*. *casei* strains. Moreover, potential S-layer proteins were only found in the *L*. *brevis* 1595 genome, whereas genes potentially involved in capsular polysaccharide biosynthesis were present in *L*. *casei* 1542, *L*. *lactis* 1596 and *L*. *plantarum* strains. All strains were also found to possess one or more proteins annotated as Internalin J precursor. However, the conserved domain content strongly varies between these proteins (S3 Table).

The presence of potential antibiotic resistance genes was checked, revealing only a few potential antibiotic resistance genes (S4 Table). Indeed, no gene encoding antibiotic resistance genes was found in *L*. *brevis* 1595. All strains but one (*L*. *brevis* 1595) encode a potential bacitracin resistance gene. Finally, *L*. *lactis* 1596 was found to carry two additional genes potentially coding for fluoroquinolone and tetracycline resistance.

## Discussion

### Selection of potential probiotic strains

In this study, we isolated 165 clones from bovine mammary microbiota and did an in-depth characterization of ten non-redundant LAB strains with regard to their beneficial properties in a mammary gland context. During this sampling campaign, we isolated a majority of enterococci (~29%: *E*. *faecium* and *E*. *hirae*) and streptococci (~29%: *S*. *infantarius* and *S*. *uberis*). These species have been previously isolated from foremilk samples and are reportedly associated with mastitis and fecal contamination [8]. Of note, in our study, enterococci and streptococci were also mainly isolated from foremilk and after growth on M17. We thus focused our study on LAB strains belonging to *Lactococcus* spp. (6.6% of the isolates) and *Lactobacillus* spp. (22.4%) genera since they have a GRAS status and are already used as probiotics in specific contexts.

As beneficial properties, we characterized LAB capacity to colonize mammary epithelial cells *in vitro*, their inhibitory potential on three important bovine mastitis pathogens, and their capacity to stimulate the innate immune system. In addition, the genomes of five of these ten strains were fully sequenced, revealing interesting genetic determinants potentially linked to the phenotypes observed and allowing potential utilization of strains devoid of antibioresistance determinants. The five selected strains harbored only a few antibiotic resistance genes. All strains but one (*L*. *brevis* 1595) show resistance to bacitracin, whereas *L*. *lactis* presented two additional resistance determinants against tetracycline and fluoroquinolone, antibiotics commonly indicated for intramammary or parenteral mastitis treatment in European countries (S3 Table) [30–32]. Such a large screening of beneficial properties as well as of potential undesirable genetic markers had never been done in this context before and allowed us to identify LAB candidates that exhibited one or more promising properties for the prevention and/or treatment of mastitis.

### Competition for the niche

One important trait for probiotics is the ability to compete with pathogens for niche colonization. We thus characterized the LAB strains with regard to their inhibitory potentials in terms of acidification, production of hydrogen peroxide or other inhibitory compounds such as bacteriocin-like compounds and with regard to their colonization capacity. The ability of the ten LAB isolates to inhibit the growth of representative strains of *E*. *coli*, *S*. *uberis* and *S*. *aureus* was highly species-specific. The inhibition observed likely relied on acidification since neutralization of the supernatants totally relieved their inhibitory activity. Supernatants of *L*. *plantarum* and *L*. *casei* strains showed the highest inhibitory potential in relation to the lowest pH values. However, we cannot totally exclude the production of inhibitory compounds such as bacteriocins which would be more active at low pH. Of note, in mammary gland, pH is close to neutrality -respectively 6.89 and 6.71 for foremilk and stripping milk [33]. The impact of organic acid production by LAB or other antibacterial compounds, which would be active at low pH, would thus be limited. None of our ten LAB strains were found to produce hydrogen peroxide. Hydrogen peroxide production is considered to be an interesting trait of vaginal probiotic LAB [34]. It was also previously reported for some (but not all) LAB strains isolated from mammary microbiota, although with a lower frequency than in vaginal LAB [8,35]. This might reflect a different adaptation of LAB strains within mammary and vaginal ecosystems.

The ability to colonize tissues and, as a result, exert a prolonged beneficial effect and or compete with pathogens for the niche is one of the criteria used to select a candidate probiotic strain. Our results showed that adhesion capacities of LAB vary between strains. *L*. *brevis* 1595 and 1597 and *L*. *plantarum* 1610 harbored the strongest adhesion to bMEC. These strains were also those that had the highest hydrophobicity. Such correlation between adhesion and hydrophobicity had previously been reported [8]. One strain, namely *L*. *brevis* 1597, also exhibited autoaggregation properties. Autoaggregation is thought to favor formation of protective biofilm [35]. Autoaggregative strains may also titer pathogenic microorganisms by coaggregation and facilitate their clearance [36].

Genomic comparison of strains exhibiting high (*L*. *brevis* 1595 and *L*. *plantarum* 1610) or low adhesion rates (*L*. *plantarum* 1612, *L*. *lactis* 1596, *L*. *casei* 1542 and BL23) (S3 Table) revealed differences in terms of genes potentially involved in host tissue colonization. Such variations in adhesion determinants have already been reported in LAB and include several proteins directly involved in adhesion to mucus, fibronectin or collagen, S-layer proteins or proteins involved in capsular polysaccharide biosynthesis, as well as some house-keeping gene products [6,37,38]. In our case, three to four MucBP domain proteins were found in strains exhibiting high adhesion rates, whereas only one MucBP domain protein was found in *L*. *lactis* 1596 and none were present in *L*. *casei* genomes. Moreover, only *L*. *brevis* 1595 encodes S-layer proteins and the presence of capsular polysaccharide biosynthesis genes was also strain-dependent. Moreover, it is noticeable that *L*. *brevis* 1595 and the two *L*. *plantarum* strains, possess two potential fibronectin-binding proteins, carrying the conserved domains FNB and FnbA, respectively, whereas the low adhesive strains, except *L*. *plantarum* 1612, only possess one copy of FbpA-domain protein. Whether the variations in adhesion capacities we observed in this study are linked to the presence or absence of one or several of the above-mentioned adhesion determinants remains to be explored.

Studies investigating LAB colonization capacity are generally restricted to the evaluation of their adhesion properties, with the aim to prevent tissue colonization by pathogens. The capacity to internalize and, possibly, to survive and proliferate within cells is classically associated with pathogenic bacteria and, as a result, not explored for the so-called “beneficial bacteria”. Only a few studies report LAB internalization, with the aim to use LAB as vehicles for intracellular delivery of molecules [39]. In this study, LAB capacity to internalize into host cells was investigated, showing strain-dependence. This ability was related to the ability to adhere, as illustrated by a Pearson correlation coefficient of 0.89 between both capacities at a MOI of 400:1. Interestingly, full genome sequencing of the five strains revealed the presence of Internalin J-like proteins, which share conserved domains with *Listeria monocytogenes* Internalin J [40]. Of note, despite similarities with Internalin A and B, the exact function of Internalin J in *L*. *monocytogenes* virulence is not yet fully understood [40]. The internalization capacity of the tested LAB was limited compared to the one of two major etiologic agents of mastitis (*S*. *aureus* and *E*. *coli*): internalized LAB population was similar to internalized *S*. *aureus* and *E*. *coli* populations using similar assays, but with a higher MOI for LAB compared to *S*. *aureus* and *E*. *coli* (MOI of 400:1 and 2000:1 for LAB and MOI of 100:1 and 10:1 for *S*. *aureus* and *E*. *coli* respectively) [12,41].Nevertheless, it raises questions about the possible persistence of LAB in tissue and their effect on cellular physiology, cell cycle or epigenetic modifications, as observed with pathogenic bacteria such as *S*. *aureus* [42]. This question requires further investigation.

### Immunomodulation by bovine mammary LAB strains

On the one hand, a probiotic LAB candidate with a slight pro-inflammatory capacity can be of interest to stimulate innate immunity and to thus prevent mastitis. On the other hand, a strain with an anti-inflammatory capacity would help resolve the inflammation in infectious mastitis and help the return to lactation. In this study, LAB did not significantly alter IL-8 production by bMEC (PS line) when incubated alone with cells. In contrast, most LAB exhibited anti-inflammatory properties on *E*. *coli*-stimulated bMEC, where *L*. *brevis* 1595, 1597 and *L*. *casei* 1542 were the most efficient with a ~three-fold decrease of IL-8 production. We first attempted to evaluate the immunomodulation properties of LAB on another well-known model, i.e., HT-29, which has been widely used to evaluate these properties for LAB strains dedicated to gut. Using this model, LAB did not exhibit similar immunomodulation properties (S2 Fig). Such a discrepancy underlines the importance of using a cellular model relevant for the ecosystem addressed. Some of the above-mentioned determinants that are putatively involved in adhesion and internalization have also been correlated with the LAB immunomodulation properties [37]. The involvement of these determinants in the immunomodulation properties will deserve further experiments.

In conclusion, we have selected a set of LAB strains isolated from the bovine mammary gland based on their PFGE pattern and screened them for their potential to colonize mammary gland tissue and for their immunomodulation properties. Some strains present high *in vitro* adhesion and invasion capacity and are potential candidates able to compete with pathogens for the colonization of mammary gland tissue and to exert prolonged beneficial effects. Pro-inflammatory properties could help stimulate the innate immune system and promote the clearance of pathogens. Anti-inflammatory properties could contribute to the decrease of inflammation in association with or following antibiotic treatment. The presence of undesirable genetic elements such as antibiotic resistance genes was also checked in order to prevent the risk of dissemination of antibiotic resistance determinants. These candidate strains require further investigation to evaluate their barrier effect with regard to major mastitis pathogens and their immune-modulatory potential on bovine mammary epithelial cells. The mandatory experiments will be to assess *in vivo* safety and to challenge their efficacy in field conditions.

## Supporting Information

S1 Fig Dendograms of PFGE patterns of *Lactobacillus* sp. (A) obtained with endonuclease AscI and *Lactococcus* sp. (B) obtained with endonuclease SmaI. The similarities of the profiles were calculated using Dice's coefficient and dendograms were obtained by the UPGMA clustering algorithm.
(TIF)

S2 Fig Modulation of cytokine IL-8 production by the HT-29 cell line in the presence of LAB isolates. Bars represent the mean IL-8 production ± standard deviation for three independent assays, normalized with regard to IL-8 production when stimulation of the HT-29 cell line was done with TNF-α alone (reference condition). Differences in IL-8 production with regard to the reference condition were assessed using the Mann-Whitney test (# p < 0.1).
(TIF)

S1 Table Sequencing statistics. (DOCX)

S2 Table Bacterial diversity of isolates from the bovine mammary gland. Total number of isolates is indicated for each species/genus as well as the source of the isolates for each species (i.e. cytobrush of teat canal or fore-milk sample) and the medium from which the isolates for each species were selected.
(DOCX)

S3 Table Potential bacterial determinants of LAB colonization capacities and immunomodulation properties in *L*. *brevis* 1595, *L*. *casei* 1542, *L*. *lactis* 1696, *L*. *plantarum* 1610 and 1612 and *L*. *casei* BL23. (DOCX)

S4 Table Potential antibiotic resistance genes encoded in *L*. *brevis* 1595, *L*. *casei* 1542, *L*. *lactis* 1696, *L*. *plantarum* 1610 and 1612 and *L*. *casei* BL23. (DOCX)
