# Supplementary figures and images for: Lactic Acid Bacteria Isolated from Bovine Mammary Microbiota: Potential Allies against Bovine Mastitis
Source: PLoS One. 2015 Dec 29;10(12):e0144831. doi: 10.1371/journal.pone.0144831 (PMC4694705; doi:10.1371/journal.pone.0144831)

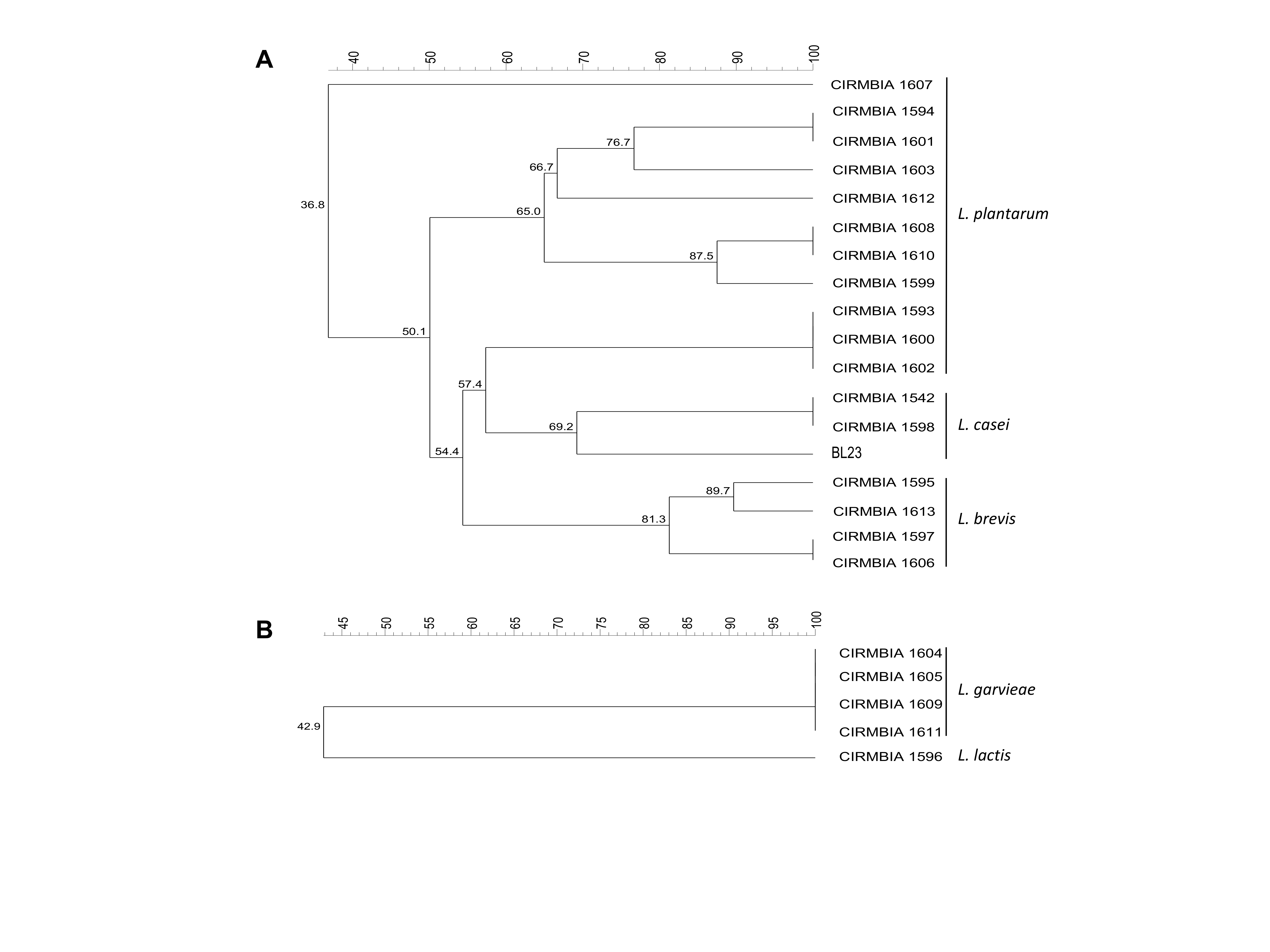

Supplement: S1 Fig — (A) obtained with endonuclease AscI and Lactococcus sp. (B) obtained with endonuclease SmaI. The similarities of the profiles were calculated using Dice's coefficient and dendograms were obtained by the UPGMA clustering algorithm. (TIF) [file pone.0144831.s001.tif]

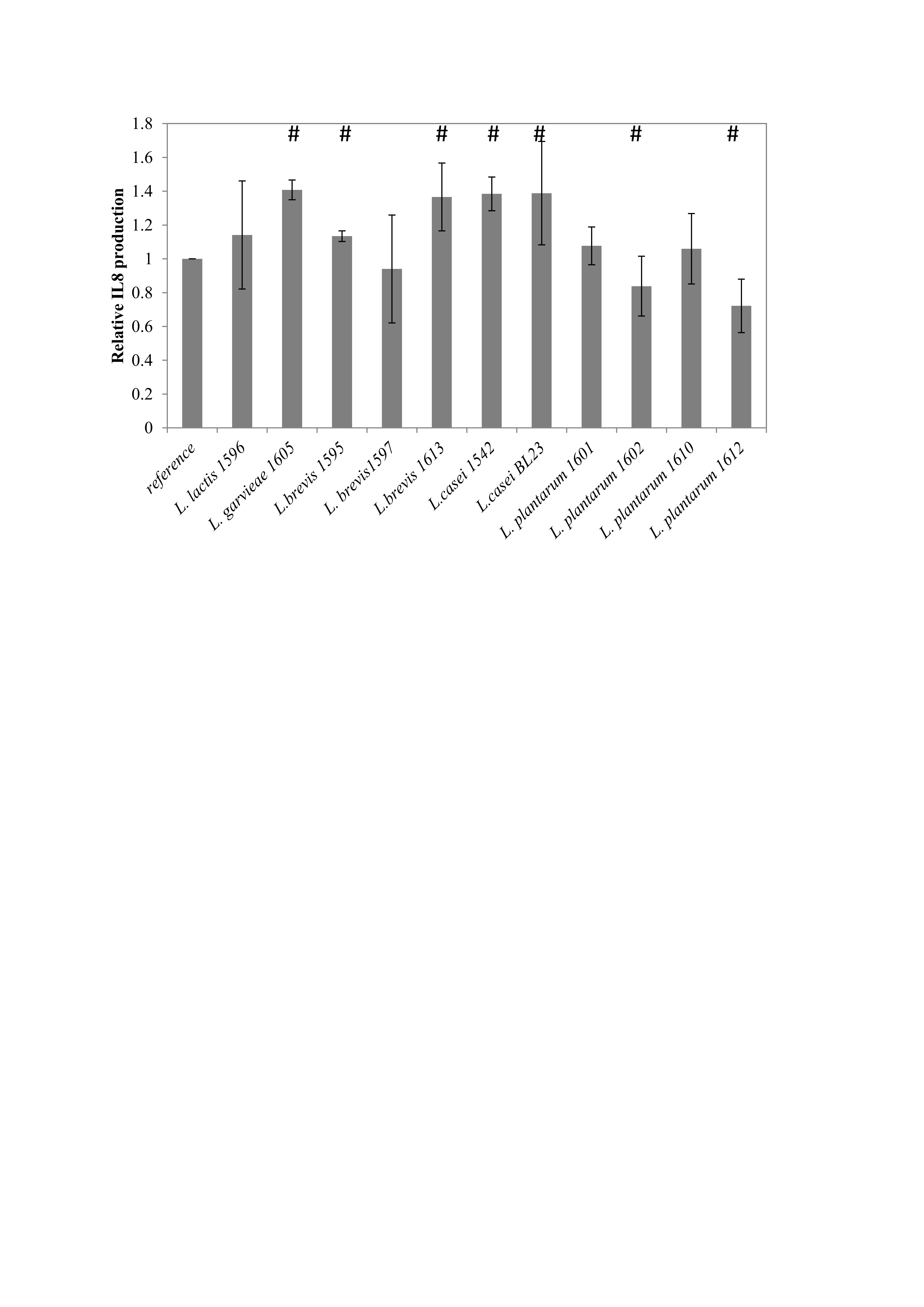

Supplement: S2 Fig — Bars represent the mean IL-8 production ± standard deviation for three independent assays, normalized with regard to IL-8 production when stimulation of the HT-29 cell line was done with TNF-α alone (reference condition). Differences in IL-8 production with regard to the reference condition were assessed using the Mann-Whitney test (# p < 0.1). (TIF) [file pone.0144831.s002.tif]
