# Supplementary material for: Lactic Acid Bacteria Isolated from Bovine Mammary Microbiota: Potential Allies against Bovine Mastitis
Source: PLoS One. 2015 Dec 29;10(12):e0144831. doi: 10.1371/journal.pone.0144831 (PMC4694705; doi:10.1371/journal.pone.0144831)
Supplement: S1 Table — (DOCX) [file pone.0144831.s003.docx]

**S1 Table.** Sequencing statistics

| **Contig Statistics** | | | | | | | | | |  | **Raw Read Information** | |
| --- | --- | --- | --- | --- | --- | --- | --- | --- | --- | --- | --- | --- |
| strain |  | n | min | median | mean | N50 | max | sum | GC |  | Nb of Reads | Th. Coverage |
|  |  |  | [bp] | [bp] | [kbp] | [kbp] | [kbp] | [Mbp] | [%] |  | [-] | (Approx) |
| *Lactobacillus brevis* 1595 | Lb1595 | 50 | 538 | 24 | 49 | 120 | 257 | 2.44 | 45.80 |  | 20410436 | 1250x |
| *Lactobacillus casei* 1542 | Lc1542 | 86 | 569 | 11 | 34 | 98 | 265 | 2.92 | 46.50 |  | 17923669 | 920x |
| *Lactococcus lactis* 1596 | Ll1596 | 35 | 976 | 28 | 68 | 211 | 287 | 2.39 | 35.00 |  | 24179903 | 1500x |
| *Lactobacillus plantarum* 1610 | Lp1610 | 27 | 539 | 41 | 122 | 277 | 635 | 3.30 | 44.40 |  | 21127493 | 950x |
| *Lactobacillus plantarum* 1612 | Lp1612 | 25 | 515 | 61 | 130 | 328 | 715 | 3.24 | 44.40 |  | 20991389 | 970x |
