# Supplementary material for: Lactic Acid Bacteria Isolated from Bovine Mammary Microbiota: Potential Allies against Bovine Mastitis
Source: PLoS One. 2015 Dec 29;10(12):e0144831. doi: 10.1371/journal.pone.0144831 (PMC4694705; doi:10.1371/journal.pone.0144831)
Supplement: S2 Table — Total number of isolates is indicated for each species/genus as well as the source of the isolates for each species (i.e. cytobrush of teat canal or fore-milk sample) and the medium from which the isolates for each species were selected. (DOCX) [file pone.0144831.s004.docx]

**S2 Table. Bacterial diversity of isolates from bovine mammary gland**

Total number of isolates is indicated for each species/genus as well as the source of the isolates for each species (i.e. cytobrush of teat canal or fore-milk sample) and the medium from which the isolates for each species were selected.

|  | **Total number of isolates** | **%** | **Number of isolates on** | | | **Source** | |
| --- | --- | --- | --- | --- | --- | --- | --- |
|  |  |  | **M17** | **MRS** | **MRS 5.4** | **Fore-milk** | **cytobrush** |
| **enterobacteria** | **7** | **9.2%** | **7** |  |  | **4** | **3** |
| *Escherichia coli* | 6 | 7.9% | 6 |  |  | 3 | 3 |
| *Klebsiella oxytoca* | 1 | 1.3% | 1 |  |  | 1 |  |
| **enterococci** | **22** | **28.9%** | **13** | **6** | **3** | **16** | **6** |
| *Enterococcus faecalis* | 1 | 1.3% | 1 |  |  |  | 1 |
| *Enterococcus faecium* | 7 | 9.2% | 3 | 3 | 1 | 7 |  |
| *Enterococcus hirae* | 4 | 5.3% | 2 | 1 | 1 | 2 | 2 |
| *Enterococcus sp* | 10 | 13.2% | 7 | 2 | 1 | 7 | 3 |
| **lactobacilli** | **17** | **22.4%** | **1** | **5** | **11** | **8** | **9** |
| *Lactobacillus brevis* | 4 | 5.3% |  |  | 4 | 2 | 2 |
| *Lactobacillus casei* | 2 | 2.6% |  |  | 2 | 0 | 2 |
| *Lactobacillus plantarum* | 11 | 14.5% | 1 | 5 | 5 | 6 | 5 |
| **lactococci** | **5** | **6.6%** | **4** | **1** |  | **2** | **3** |
| *Lactococcus garvieae* | 4 | 5.3% | 3 | 1 |  | 1 | 3 |
| *Lactococcus lactis* | 1 | 1.3% | 1 |  |  | 1 |  |
| **staphylococci** | **3** | **3.9%** | **3** |  |  | **3** |  |
| *Staphylococcus aureus* | 1 | 1.3% | 1 |  |  | 1 |  |
| *Staphylococcus sp* | 2 | 2.6% | 2 |  |  | 2 |  |
| **streptococci** | **22** | **28.9%** | **18** | **4** |  | **15** | **7** |
| *Streptococcus infantarius* | 13 | 17.1% | 9 | 4 |  | 9 | 4 |
| *Streptococcus parauberis* | 1 | 1.3% | 1 |  |  | 0 | 1 |
| *Streptococcus uberis* | 4 | 5.3% | 4 |  |  | 3 | 1 |
| *Streptococcus sp* | 4 | 5.3% | 4 |  |  | 3 | 1 |
| **total** | **76** |  | **46** | **16** | **14** | **48** | **28** |
