# Supplementary material for: Lactic Acid Bacteria Isolated from Bovine Mammary Microbiota: Potential Allies against Bovine Mastitis
Source: PLoS One. 2015 Dec 29;10(12):e0144831. doi: 10.1371/journal.pone.0144831 (PMC4694705; doi:10.1371/journal.pone.0144831)
Supplement: S3 Table — (DOCX) [file pone.0144831.s005.docx]

**S3 Table.** Potential bacterial determinants of LAB colonization capacities and immunomodulation properties *in L. brevis* 1595, *L. casei* 1542*, L. lactis* 1596, *L. plantarum* 1610 and 1612 and *L. casei* BL23

| ID | Description | Conserved  Domains ^a^ | Prediction ^b^ | Length | | | signal peptide ^b^ | | | Comment ^b^ | | |  |  |
| --- | --- | --- | --- | --- | --- | --- | --- | --- | --- | --- | --- | --- | --- | --- |
| ***L. brevis* 1595** |  |  |  |  | | |  | | |  | | |  |  |
| lactobrevis_1595_01027 | Cna protein B-type domain protein | Cna-B, collagen_bind superfamily | PSE | 619 | | | Y | | | LPxTG | | |  |  |
| lactobrevis_1595_01481 | Cna protein B-type domain protein | Cna-B, collagen_bind superfamily | PSE | 663 | | | Y | | | LPxTG | | |  |  |
| lactobrevis_1595_00487 | Collagen binding domain protein | collagen_bind superfamily | PSE | 443 | | | Y | | |  | | |  |  |
| lactobrevis_1595_01650 | Internalin-J precursor | DUF285, Big_3 superfamily (Ig-like domain) | SECRETED | | 648 | | | Y | | |  | | |  |
| lactobrevis_1595_01722 | Internalin-J precursor | MucBP, LRR_4, LRR_8 | PSE | 912 | | | Y | | | LPxTG | | |  |  |
| lactobrevis_1595_01290 | MucBP domain protein |  | PSE | 422 | | | N | | |  | | |  |  |
| lactobrevis_1595_01879 | MucBP domain protein | MucBP | PSE | 1519 | | | Y | | | LPxTG | | |  |  |
| lactobrevis_1595_02463 | MucBP domain protein | MucBP | PSE | 1111 | | | Y | | | LPxTG | | |  |  |
| lactobrevis_1595_02534 | MucBP domain protein |  | PSE | 454 | | | N | | | LPxTG | | |  |  |
| lactobrevis_1595_01519 | S-layer protein precursor |  | SECRETED | 470 | | | Y | | |  | | |  |  |
| lactobrevis_1595_02073 | S-layer protein precursor |  | SECRETED | 413 | | | Y | | |  | | |  |  |
| lactobrevis_1595_01520 | S-layer protein precursor |  | PSE | 457 | | | Y | | |  | | |  |  |
| lactobrevis_1595_02440 | S-layer protein precursor |  | PSE | 427 | | | Y | | |  | | |  |  |
| lactobrevis_1595_01876 | Sortase family protein |  | PSE | 237 | | | Y | | |  | | |  |  |
| lactobrevis_1595_00365 | Fibronectin-binding protein | FBP | CYTOPLASMIC | 215 | | | N | | |  | | |  |  |
| lactobrevis_1595_00598 | Fibronectin-binding protein | FbpA | CYTOPLASMIC | 568 | | | N | | |  | | |  |  |
| ***L. casei* 1542** |  |  |  |  | | |  | | |  | | |  |  |
| lactocasei_1542_01840 | Capsular polysaccharide type 8 biosynthesis protein cap8A |  | PSE | 312 | | | N | | |  | | |  |  |
| lactocasei_1542_02809 | Capsular polysaccharide type 8 biosynthesis protein cap8A |  | PSE | 309 | | | Y | | |  | | |  |  |
| ID | Description | Conserved  Domains ^a^ | Prediction ^b^ | Length | | | signal peptide ^b^ | | | Comment ^b^ | | |  |  |
| ***L. casei* 1542** |  |  |  |  | | |  | | |  | | |  |  |
| lactocasei_1542_00868 | Cna protein B-type domain protein |  | PSE | 334 | | | Y | | | LPxTG | | |  |  |
| lactocasei_1542_00986 | Cna protein B-type domain protein | Cna-B | PSE | 1269 | | | Y | | | LPxTG | | |  |  |
| lactocasei_1542_01071 | Cna protein B-type domain protein | Cna-B | PSE | 1001 | | | Y | | | LPxTG | | |  |  |
| lactocasei_1542_01015 | Cna protein B-type domain protein | collagen_bind superfamily, Cna-B | PSE | 611 | | | Y | | |  | | |  |  |
| lactocasei_1542_02086 | Cna protein B-type domain protein | Cna-B | PSE | 2724 | | | Y | | |  | | |  |  |
| lactocasei_1542_01073 | Fimbrial subunit type 1 precursor | Cna-B | PSE | 519 | | | Y | | | LPxTG | | |  |  |
| lactocasei_1542_01105 | Internalin-J precursor |  | SECRETED | 192 | | | Y | | |  | | |  |  |
| lactocasei_1542_00689 | Internalin-J precursor |  | PSE | 230 | | |  | | | LPxTG | | |  |  |
| lactocasei_1542_02428 | Sortase family protein |  | SECRETED | 296 | | | Y | | |  | | |  |  |
| lactocasei_1542_00867 | Sortase family protein |  | PSE | 358 | | | Y | | |  | | |  |  |
| lactocasei_1542_01074 | Sortase family protein |  | PSE | 275 | | | Y | | |  | | |  |  |
| lactocasei_1542_02561 | Sortase family protein |  | PSE | 233 | | |  | | |  | | |  |  |
| lactocasei_1542_00870 | von Willebrand factor type A domain protein | Cna-B, collagenBindB superfamily | PSE | 909 | | | Y | | | LPxTG | | |  |  |
| lactocasei_1542_00633 | Hypothetical protein (FbpA domain) | FbpA | CYTOPLASMIC | 567 | | | N | | |  | | |  |  |
| ***L. lactis* 1596** |  |  |  |  | | |  | | |  | | |  |  |
| lactolactis_1596_00950 | Capsular polysaccharide type 8 biosynthesis protein cap8A |  | PSE | 259 | | | Y | | |  | | |  |  |
| lactolactis_1596_00957 | Capsular polysaccharide type 8 biosynthesis protein cap8A |  | PSE | 191 | | | N | | |  | | |  |  |
| lactolactis_1596_01025 | Cna protein B-type domain protein | Collagen_bind, Cna-B | PSE | 1983 | | | Y | | | LPxTG | | |  |  |
| lactolactis_1596_01964 | Cna protein B-type domain protein |  | PSE | 614 | | | Y | | | LPxTG | | |  |  |
| lactolactis_1596_00248 | Collagen adhesin precursor | collagenBindB superfamily | PSE | | | 822 | | | Y | | |  | | |
| lactolactis_1596_01240 | Collagen adhesin precursor | collagen_bind, collagen_ BindB superfamily | PSE | 366 | | | N | | | LPxTG | | |  |  |
| lactolactis_1596_2132 | Serine-rich adhesin for platelets precursor | MucBP | PSE | 584 | | | Y | | | LPxTG | | |  |  |
| ID | Description | Conserved  Domains ^a^ | Prediction ^b^ | Length | | | signal peptide ^b^ | | | Comment ^b^ | | |  |  |
| ***L. lactis* 1596** |  |  |  |  | | |  | | |  | | |  |  |
| lactolactis_1596_02063 | Internalin-J precursor |  | SECRETED | 338 | | | Y | | |  | | |  |  |
| lactolactis_1596_00327 | Sortase family protein |  | PSE | 248 | | | Y | | |  | | |  |  |
| lactolactis_1596_01963 | Sortase family protein |  | PSE | 431 | | | Y | | |  | | |  |  |
| lactolactis_1596_02139 | Fibronectin-binding protein | FBP superfamily | CYTOPLASMIC | 218 | | | N | | |  | | |  |  |
| ***L. plantarum* 1610** |  |  |  |  | | |  | | |  | | |  |  |
| lactoplantarum_1610_02069 | Capsular polysaccharide phosphotransferase cps12A |  | PSE | 321 | | | N | | |  | | |  |  |
| lactoplantarum_1610_00571 | Capsular polysaccharide type 8 biosynthesis protein cap8A |  | PSE | 252 | | | N | | |  | | |  |  |
| lactoplantarum_1610_03115 | Capsular polysaccharide type 8 biosynthesis protein cap8A |  | PSE | 256 | | | Y | | |  | | |  |  |
| lactoplantarum_1610_02763 | Cna protein B-type domain protein | collagen_bind superfamily, Cna-B | PSE | 647 | | | Y | | |  | | |  |  |
| lactoplantarum_1610_01581 | Collagen binding domain protein | collagen_bind superfamily | PSE | 617 | | | Y | | |  | | |  |  |
| lactoplantarum_1610_01448 | Internalin-J precursor | Liste_lipo_26, DUF285 | SECRETED | 750 | | | Y | | |  | | |  |  |
| lactoplantarum_1610_01441 | Internalin-J precursor | LRR_4, LRR_8 | PSE | 1189 | | | Y | | | LPxTG | | |  |  |
| lactoplantarum_1610_01827 | Internalin-J precursor |  | PSE | 1260 | | | Y | | | LPxTG | | |  |  |
| lactoplantarum_1610_02840 | Internalin-J precursor |  | PSE | 906 | | | Y | | | LPxTG | | |  |  |
| lactoplantarum_1610_00178 | MucBP domain protein | MucBP, MucBP superfamily | PSE | 2217 | | | Y | | | LPxTG | | |  |  |
| lactoplantarum_1610_01451 | MucBP domain protein | MucBP, MucBP superfamily | PSE | 2023 | | | Y | | | LPxTG | | |  |  |
| lactoplantarum_1610_03092 | MucBP domain protein | MucBP, MucBP superfamily | PSE | 252 | | |  | | | LPxTG | | |  |  |
| lactoplantarum_1610_01497 | IgA FC receptor precursor | MucBP superfamily | PSE |  | | | Y | | | LPxTG | | |  |  |
| lactoplantarum_1610_00912 | Agglutinin receptor precursor | collagenBindB superfamily | PSE | 994 | | | Y | | |  | | |  |  |
| lactoplantarum_1610_00657 | Sortase family protein |  | SECRETED | 234 | | | Y | | |  | | |  |  |
|  |  |  |  |  | | |  | | |  | | |  |  |
| ID | Description | Conserved  Domains ^a^ | Prediction ^b^ | Length | | | signal peptide ^b^ | | | Comment ^b^ | | |  |  |
| ***L. plantarum* 1610** |  |  |  |  | | |  | | |  | | |  |  |
| lactoplantarum_1610_01031 | Fibronectin-binding protein | FBP, FBP superfamily | CYTOPLASMIC | 215 | | | N | | |  | | |  |  |
| lactoplantarum_1610_00314 | Hypothetical protein (FbpA domain) | FbpA | CYTOPLASMIC | 568 | | | N | | |  | | |  |  |
| ***L. plantarum* 1612** |  |  |  |  | | |  | | |  | | |  |  |
| lactoplantarum_1612_02954 | Capsular polysaccharide type 8 biosynthesis protein cap8A |  | PSE | 255 | | | N | | |  | | |  |  |
| lactoplantarum_1612_03033 | Capsular polysaccharide type 8 biosynthesis protein cap8A |  | PSE | 256 | | | N | | |  | | |  |  |
| lactoplantarum_1612_00297 | Agglutinin receptor precursor | collagenBindB superfamily, Cna-B | PSE | 1038 | | | Y | | |  | | |  |  |
| lactoplantarum_1612_1695 | Agglutinin receptor precursor | Cna-B, collagen_bind superfamily | PSE | 724 | | | Y | | | LPxTG | | |  |  |
| lactoplantarum_1612_02566 | Collagen binding domain protein | collagen_bind superfamily | PSE | 617 | | | Y | | |  | | |  |  |
| lactoplantarum_1612_01701 | Immunoglobulin G-binding protein A precursor | collagen_bind superfamily | PSE | 554 | | | Y | | | LPxTG | | |  |  |
| lactoplantarum_1612_02471 | Internalin-J precursor | LRR_4,LRR_8 | PSE | 1194 | | | Y | | | LPxTG | | |  |  |
| lactoplantarum_1612_02415 | IgA FC receptor precursor (MucBP domain) | MucBP, MucBP superfamily | PSE | 1369 | | | Y | | | LPxTG | | |  |  |
| lactoplantarum_1612_01057 | MucBP domain protein | MucBP, MucBP superfamily | PSE | 2217 | | | Y | | | LPxTG | | |  |  |
| lactoplantarum_1612_02461 | MucBP domain protein | MucBP, MucBP superfamily | PSE | 2032 | | | Y | | | LPxTG | | |  |  |
| lactoplantarum_1612_00039 | Sortase family protein |  | SECRETED | 234 | | | Y | | |  | | |  |  |
| lactoplantarum_1612_00426 | Fibronectin-binding protein | FBP, FBP superfamily | CYTOPLASMIC | 215 | | | N | | |  | | |  |  |
| lactoplantarum_1612_00918 | Hypothetical protein (FbpA domain) | FbpA | CYTOPLASMIC | 568 | | | N | | |  | | |  |  |
| ***L. casei* BL23** |  |  |  |  | | |  | | |  | | |  |  |
| gi\|191637338\|ref\|YP_001986504.1\| | hypothetical protein LCABL_05200 | Cna-B,collagenBindB superfamily | PSE | 909 | | | Y | | | LPxTG | | |  |  |
| gi\|191639298\|ref\|YP_001988464.1\| | hypothetical protein LCABL_25400 | Cna-B | PSE | 519 | | | Y | | | LPxTG | | |  |  |
| gi\|191639300\|ref\|YP_001988466.1\| | outer membrane protein | Cna-B | PSE | 1001 | | | Y | | | LPxTG | | |  |  |
|  |  |  |  |  | | |  | | |  | | |  |  |
| ID | Description | Conserved  Domains ^a^ | Prediction ^b^ | Length | | | signal peptide ^b^ | | | Comment ^b^ | | |  |  |
| ***L. casei* BL23** |  |  |  |  | | |  | | |  | | |  |  |
| gi\|191639361\|ref\|YP_001988527.1\| | outer membrane protein | Cna-B,collagen_bind superfamily | PSE | 611 | | | Y | | |  | | |  |  |
| gi\|191639436\|ref\|YP_001988602.1\| | outer membrane protein | Cna-B | PSE | 2726 | | | Y | | |  | | |  |  |
| gi\|191639856\|ref\|YP_001989022.1\| | Adhesion exoprotein |  | PSE | 672 | | | N | | | LPxTG | | |  |  |
| gi\|191637480\|ref\|YP_001986646.1\| | Collagen binding protein | SBP-bac-3 | PSE | 270 | | | Y | | |  | | |  |  |
| gi\|191637340\|ref\|YP_001986506.1\| | Fimbriae subunit |  | PSE | 334 | | | Y | | | LPxTG | | |  |  |
| gi\|191639262\|ref\|YP_001988428.1\| | Internalin-J |  | PSE | 423 | | | Y | | | LPxTG | | |  |  |
| gi\|191637433\|ref\|YP_001986599.1\| | sortase srtA2 (LCABL_06160) |  | SECRETED | 223 | | | Y | | |  | | |  |  |
| gi\|191639079\|ref\|YP_001988245.1\| | sortase srtA1 (LCABL_23200) |  | PSE | 233 | | | N | | |  | | |  |  |
| gi\|191639297\|ref\|YP_001988463.1\| | sortase srtC1 (LCABL_25390) |  | PSE | 275 | | | Y | | |  | | |  |  |
| gi\|191637341\|ref\|YP_001986507.1\| | sortase srtC2 (LCABL_05230) |  | PSE | 358 | | | Y | | |  | | |  |  |
| gi\|191638435\|ref\|YP_001987601.1\| | Fibronectin-binding protein A | FbpA | CYTOPLASMIC | 567 | | | N | | |  | | |  |  |

^a^ as determined using conserved domain database search tool. pfam06458 (MucBP): mucine-binding protein domain; cl05785: MucBP superfamily; pfam05737 (Collagen_bind): Collagen binding domain; pfam05738 (Cna-B): Cna protein B-type domain (this domain is found in *Staphylococcus aureus* collagen-binding surface protein. However, this region does not mediate collagen binding); cl15753 (collagenBindB superfamily); (collagen_bind superfamily); pfam07299 (FBP) :fibronectin-binding domain; pfam05833 (FbpA): Fibronectin-binding protein A N-terminus; cl06363: FBP superfamily; pfam00497 (SBP-bac-3):bacterial extracellular solute binding proteins, family 3; pfam12799 (LRR_4): leucine rich repeat domain; pfam13855 (LRR_8); pfam03382 (DUF285): distantly related to LRR; cl06524 (Big_3 superfamily):bacterial Ig-like domain; TIGR02167 (Liste_lipo_26): bacterial surface protein 26-residue repeat, this model describes a tandem peptide repeat sequence of 25 or 26 residues, found in predicted surface proteins (often lipoproteins) from *Listeria monocytogenes, L. innocua, Enterococcus faecalis, Lactobacillus plantarum, Mycoplasma mycoides, Helicobacter hepaticus,* and other species.

^b^ as determined using SurfG software; PSE: potentially surface exposed; Y: yes; N:no; LPxTG: cell wall anchoring motif
