# Supplementary material for: Lactic Acid Bacteria Isolated from Bovine Mammary Microbiota: Potential Allies against Bovine Mastitis
Source: PLoS One. 2015 Dec 29;10(12):e0144831. doi: 10.1371/journal.pone.0144831 (PMC4694705; doi:10.1371/journal.pone.0144831)
Supplement: S4 Table — (DOCX) [file pone.0144831.s006.docx]

**S4 Table.** Potential antibiotic resistance genes encoded in *L. brevis* 1595, *L. casei* 1542, *L. lactis* 1696*, L. plantarum* 1610 and 1612 and *L. casei* BL23

| strain | locus | Type | Definition | Resistance |
| --- | --- | --- | --- | --- |
| *L. casei* 1542 | [lactocasei_1542_00040](http://ardb.cbcb.umd.edu/cgi/blast/genomecomp.cgi#lactocasei_1542_00040) | [baca](http://ardb.cbcb.umd.edu/cgi/ssquery.cgi?db=T&gn=baca) | Undecaprenyl pyrophosphate phosphatase, which consists in the sequestration of Undecaprenyl pyrophosphate. | bacitracin |
| *L. lactis* 1596 | [lactolactis_1596_02272](http://ardb.cbcb.umd.edu/cgi/blast/genomecomp.cgi#lactolactis_1596_02272) | [baca](http://ardb.cbcb.umd.edu/cgi/ssquery.cgi?db=T&gn=baca) | Undecaprenyl pyrophosphate phosphatase, which consists in the sequestration of Undecaprenyl pyrophosphate. | bacitracin |
|  | [lactolactis_1596_01002](http://ardb.cbcb.umd.edu/cgi/blast/genomecomp.cgi#lactolactis_1596_01002) | [emea](http://ardb.cbcb.umd.edu/cgi/ssquery.cgi?db=T&gn=emea) | Major facilitator superfamily transporter. Multidrug resistance efflux pump. | fluoroquinolone |
|  | [lactolactis_1596_02421](http://ardb.cbcb.umd.edu/cgi/blast/genomecomp.cgi#lactolactis_1596_02421) | [tets](http://ardb.cbcb.umd.edu/cgi/ssquery.cgi?db=T&gn=tets) | Ribosomal protection protein, which protects ribosome from the translation inhibition of tetracycline. | tetracycline |
| *L. plantarum* 1610 | [lactoplantarum_1610_01248](http://ardb.cbcb.umd.edu/cgi/blast/genomecomp.cgi#lactoplantarum_1610_01248) | [baca](http://ardb.cbcb.umd.edu/cgi/ssquery.cgi?db=T&gn=baca) | Undecaprenyl pyrophosphate phosphatase, which consists in the sequestration of Undecaprenyl pyrophosphate. | bacitracin |
| *L. plantarum* 1612 | [lactoplantarum_1612_02766](http://ardb.cbcb.umd.edu/cgi/blast/genomecomp.cgi#lactoplantarum_1612_02766) | [baca](http://ardb.cbcb.umd.edu/cgi/ssquery.cgi?db=T&gn=baca) | Undecaprenyl pyrophosphate phosphatase, which consists in the sequestration of Undecaprenyl pyrophosphate. | bacitracin |
| *L. casei* BL23 | YP 001986964.1 | baca | Undecaprenyl pyrophosphate phosphatase, which consists in the sequestration of Undecaprenyl pyrophosphate. | bacitracin |
